# Beam-Switching Antennas for 5G Millimeter-Wave Wireless Terminals

**DOI:** 10.3390/s23146285

**Published:** 2023-07-10

**Authors:** Khaled M. Morshed, Debabrata K. Karmokar, Karu P. Esselle, Ladislau Matekovits

**Affiliations:** 1 School of Engineering, Macquarie University, Sydney, NSW 2109, Australia; 2 UniSA STEM, University of South Australia, Mawson Lakes, Adelaide, SA 5095, Australia; 3 School of Electrical and Data Engineering, University of Technology Sydney, Ultimo, NSW 2007, Australia; karu.esselle@uts.edu.au; 4 Department of Electronics and Telecommunications, Politecnico di Torino, 10129 Turin, Italy; ladislau.matekovits@polito.it; 5 Istituto di Elettronica e di Ingegneria dell’Informazione e Delle Telecomunicazioni, National Research Council of Italy, 10129 Turin, Italy; 6 Department of Measurements and Optical Electronics, Politehnica University Timisoara, 300006 Timisoara, Romania

**Keywords:** millimeter-wave, 5G, 28 GHz band, leaky-wave antenna (LWA), wireless terminal, pattern reconfigurable

## Abstract

Beam-switching is one of the paramount focuses of 28 GHz millimeter-wave 5G devices. In this paper, a one-dimensional (1D) pattern reconfigurable leaky-wave antenna (LWA) was investigated and developed for wireless terminals. In order to provide a cost-effective solution, a uniform half-width LWA was used. The 1D beam-switching LWA was designed using three feed points at three different positions; by selecting the feeds, the direction of the beam can be switched. The antenna can switch the beam in three different directions along the antenna axis, such as backward, broadside, and forward. The 1D beam-switching antenna was fabricated, and because of the wide beamwidth, the measured radiation patterns can fill 128${}^{\circ }$ of space (3 dB coverage), from θ = −64${}^{\circ }$ to +64${}^{\circ }$ at ϕ = 0${}^{\circ }$. Following this, two of these antennas were placed at right angles to each other to achieve two-directional (2D) beam switching. The 2D beam-switching antenna pair was also prototyped and tested after integrating them into the ground plane of a wireless device. The antenna is able to point the beam in five different directions; moreover, its beam covers 167${}^{\circ }$ (θ = −89${}^{\circ }$ to +78${}^{\circ }$) at ϕ = 0${}^{\circ }$, and 154${}^{\circ }$ (θ = −72${}^{\circ }$ to +82${}^{\circ }$) at ϕ = 90${}^{\circ }$.

## 1. Introduction

Increasing demand for high data throughput and reliable mobile services has motivated the development of the 5G mobile communication standard [1,2,3,4,5,6,7,8,9,10,11]. Proposed millimeter-wave bands around 28 GHz have much potential for 5G mobile communications because of the availability of massive bandwidths. Five technologies that are under consideration will lead to fundamental changes in the development of future 5G mobile communications, where the aim is to achieve 100 to 1000 times the data rate of 4G [12,13,14,15,16]. These are device-centric architecture, millimeter-wave, cognitive radio, smart device, and native support for machine to machine (M2M) communications [12,14,15,17,18,19,20]. However, among these, the 28 GHz (27.5–29.5 GHz) band of millimeter-wave technology is receiving strong attention from researchers and industry due to the massive bandwidth available [13,21,22,23,24,25,26,27,28,29,30,31]. Moreover, in the United States, the Federal Communications Commission (FCC) has allocated spectrum for future 5G networks and technologies, which includes the 27.5–28.35 GHz band [32]. However, high-frequency signals experience patch loss and significant penetration loss when encountering materials such as glass, bricks, etc. [13,33,34]. An indoor wireless terminal would be useful for solving this issue.

Various antennas have been reported so far for 5G millimeter-wave communications. For example, a dual, linearly polarized antenna element with four substrate layers and three films for bonding was proposed in [2]. Then, for multi-beam radiation, a 4 × 4 Butler matrix was designed and integrated with a 1 × 4 array. The array generates four beams and covers $\pm 61^{\circ }$ and $\pm 75^{\circ }$ in x and y polarizations, respectively. A four-element antenna array with a feed network was proposed in [5]. The array radiates a fixed beam with a gain of 10.7 dBi. In [6], an aperture-sharing dual band antenna, consisting of two substrate layers, for microwave- and millimeter-wave applications was presented. The beam scanning was demonstrated through a simulation by designing a 1 × 4 array, which shows that the beam can be scanned $-45^{\circ }$ to $+45^{\circ }$ within a 3 dB scan loss. Finally, a Wilkinson power divider was designed to verify the performance of the array, which radiated the beam at $0^{\circ }$. An array of interconnected magneto-electric dipoles was proposed for 5G applications [9]. The 5×5 antenna array radiated a fixed beam with a maximum 20.44 dBi gain at 27.5 GHz. A dual-polarized microstrip patch antenna module consisting of three layers and its 1 × 4 array for 5G applications was proposed in [30]. The gain of the single element and the array were 5 and 11 dBi, respectively. In [31], a square loop antenna printed on a thin substrate with a perturbed ground plane was proposed. The gain of the antenna element and its 1 × 4 array at 28 GHz was 3.3 and 10.1 dBi, respectively. In [35], a quasi-Yagi antenna array was proposed for 28 GHz 5G cellular handsets. The antenna was designed using a 10-layer PCB stackup, and it had a parasitic and driven element, which was fed by a coplanar stripline. Two four-element arrays were designed, which were fed by a four-way T-junction power divider with a gain over 9 dBi. A beam steering phased array antenna was developed in [36] for application in 5G mobile devices with metallic casing. Two phased arrays, each consisting of eight elements, were built at the right and left edges of the metallic casing of a mobile device. The array beam scans between 0${}^{\circ }$ and 60${}^{\circ }$ with a broadside gain of 15.6 dBi (simulated). An electronically controlled leaky-wave antenna (LWA) with beam scanning at a fixed frequency was designed using an air-filled gap waveguide in [37]. Sixty patchers were used as radiating elements. In addition to the gap waveguide, two additional substrate layers were used in the antenna design. The antenna is capable of steering the beam from $-35^{\circ }$ to $+35^{\circ }$. In 5G millimeter-wave communications, it is evident that there is a strong focus on beam-switching antennas [21,36,38,39,40,41,42]. Different methods have been proposed so far, and various beam-switching antennas have been developed for 5G millimeter-wave wireless systems, as some of them discussed above. However, many of the structures are complex and require additional feed networks. Beam scanning from LWA can be achieved without an additional feed network but requires active electronic devices. It is well known that an active electronic device (e.g., a PIN diode with a direct connection to the main radiating element) suffers from significant losses at higher microwave frequencies [43,44,45]. Losses in active devices degrade the antenna’s radiation performance. Although some high-performance RF components are available, they are very expensive and will increase the cost significantly.

To avoid the above-mentioned issues and enable a cost-effective solution for wireless terminal beam-switching antennas in the millimeter-wave frequency range, a low-cost yet effective solution is presented here. The losses associated with the switching used in this work are negligible. Firstly, a 1D beam-switching antenna was designed using a single radiating element with three feed points at three different positions. Two antennas were then placed at a right angle to achieve 2D beam switching. Both 1D and 2D beam-switching antennas were fabricated and measured. The 2D beam-switching antenna has continuous 3 dB coverage from θ = −89${}^{\circ }$ to +78${}^{\circ }$ (total 167${}^{\circ }$) at ϕ = 0${}^{\circ }$, and from θ = −72${}^{\circ }$ to +82${}^{\circ }$ (total 154${}^{\circ }$) at ϕ = 90${}^{\circ }$.

## 2. Antenna Radiating Off-Broadside

The objective was to design a simple beam-switching antenna for 28 GHz 5G wireless terminals. It is known that a uniform leaky-wave antenna (LWA) scans the beam in the off-broadside region (a region between θ = 0${}^{\circ }$ and θ = 90${}^{\circ }$). Moreover, microstrip LWAs (MLWAs) are planar and low-profile antennas that can be easily integrated with millimeter-wave circuits [37,46,47,48,49,50,51,52,53,54,55,56,57,58,59,60]. Because of the desired radiation characteristics, we found that an LWA would be a simple yet effective solution for beam switching in the millimeter-wave band. This section presents the study of a half-width microstrip LWA (HW-MLWA) suitable for 28 GHz 5G wireless terminals.

### 2.1. A 28 GHz Antenna

**Figure 1:**
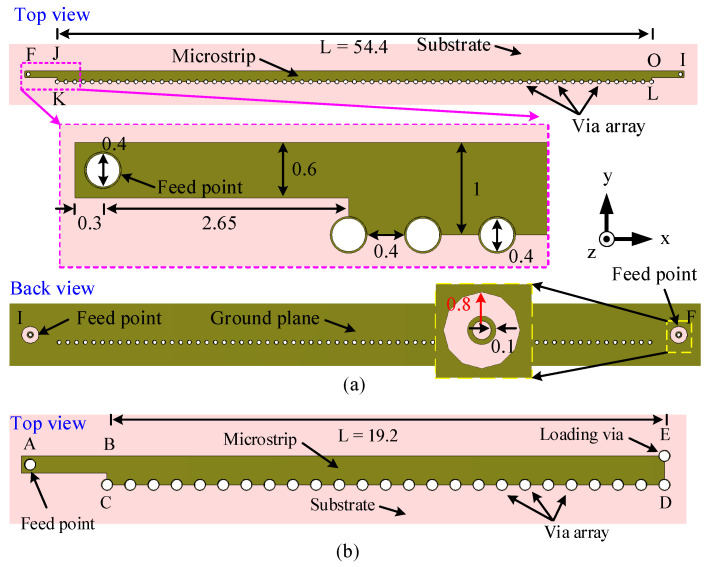
Configuration of the 28 GHz HW-MLWA with microstrip length. (**a**) *L* = 5.1$\lambda_{0}$. (**b**) *L* = 1.8$\lambda_{0}$. All dimensions are in millimeters.

One of the simplest LWAs is the HW-MLWA, which was proposed in [61] for microwave frequencies. It uses a simple microstrip line where one edge is shorted to the ground plane using a metallic wall. The metallic wall indeed supports the first higher-order mode (EH${}_{1}$) while suppressing the dominant mode (EH${}_{0}$) without any special feeding techniques. Following the method proposed in [61], an HW-MLWA was modeled first for the 28 GHz millimeter-wave band. The antenna was designed on an FR-4 substrate with dimensions of 60 mm × 8 mm, relative permittivity ($\varepsilon_{r}$) of 4.3, loss tangent (tanδ) of 0.025, and a thickness of 0.8 mm. The configuration of the antenna is shown in Figure 1a.

As shown in Figure 1a, the design starts with a microstrip JKLO with the KL edge shorted to the ground using periodically arranged shorting pins. The length (*L*) of the microstrip line is 54.4 mm or 5.1$\lambda_{0}$, where $\lambda_{0}$ is the free-space wavelength at 28 GHz. The antenna is fed using a narrow microstrip line with a width of 0.6 mm and length of 2.95 mm at the side JK. A 2.92 mm surface-mount coaxial connector is used to feed the antenna from the bottom. A similar matching line is used at the other end (OL) together with the same coaxial connector. To suppress the reflected wave, and for better impedance matching, the coaxial connector at I [Figure 1a] is terminated in a 50 Ω load. The diameter of all vias is 0.4 mm. In order to isolate the feed via from the ground plane and to ensure a guaranteed connection between the feed via and the center pin of the coaxial connector, a circular-shaped slot with an inner and outer radius of 0.8 and 0.3 mm is allowed in the ground plane as shown in the back view of Figure 1a.

**Figure 2:**
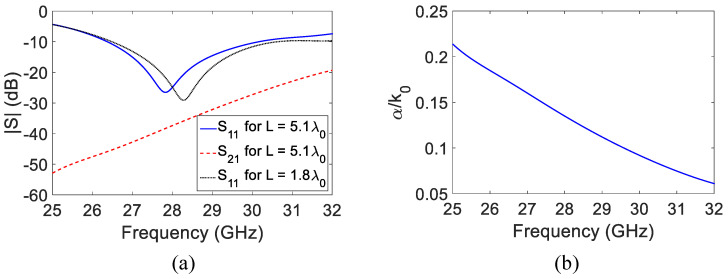
(**a**) S-parameters of two different antennas in Figure 1. (**b**) Leakage constant ($\alpha /k_{0}$) of the HW-MLWA in Figure 1a.

**Figure 3:**
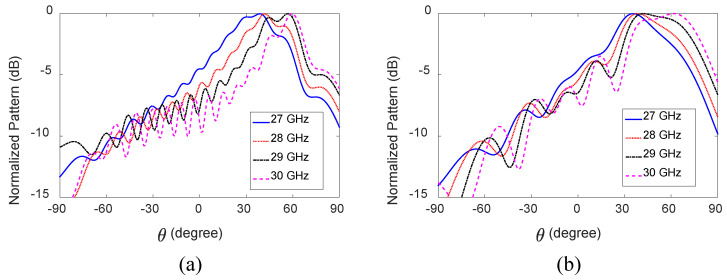
Normalized x-z plane radiation patterns of the two 28 GHz HW-MLWAs with different microstrip lengths. (**a**) *L* = 5.1$\lambda_{0}$. (**b**) *L* = 1.8$\lambda_{0}$.

The antenna in Figure 1a has a −10 dB reflection coefficient bandwidth of 3.78 GHz (26.38–30.16 GHz), as shown in Figure 2a. The predicted leakage rate ($\alpha /k_{0}$) of the HW-MLWA in Figure 1 (*L* = 5.1$\lambda_{0}$), calculated using the method presented in [62], is shown in Figure 2b. In the range of 25 to 32 GHz, the leakage rate is highest at 25 GHz; it decreases gradually and reaches a minimum value of 32 GHz. A wider beam is produced when the leakage rate ($\alpha /k_{0}$) is high, e.g., the radiation pattern is 27 GHz, as shown in Figure 3a, and a narrow beam is produced when the leakage rate ($\alpha /k_{0}$) is low, e.g., the radiation pattern is 32 GHz, as shown in Figure 3a.

**Figure 4:**
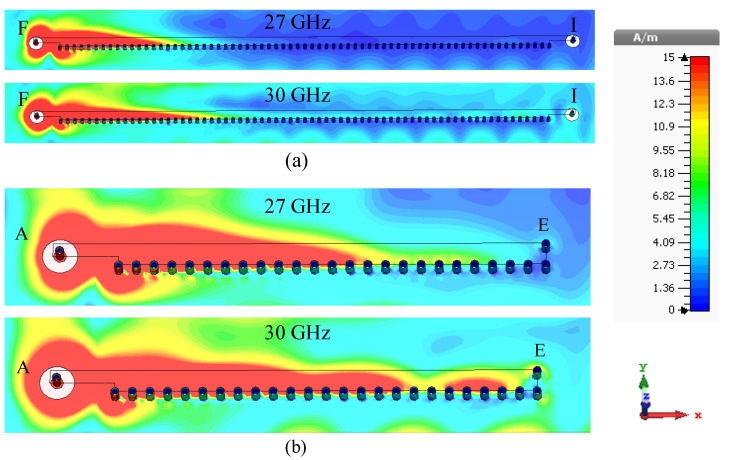
Surface current distributions (amplitude) at 27 and 30 GHz with the microstrip length. (**a**) *L* = 5.1$\lambda_{0}$. (**b**) *L* = 1.8$\lambda_{0}$.

The normalized x-z plane radiation patterns of the HW-MLWA with length *L* = 5.1$\lambda_{0}$ [Figure 1a] are shown in Figure 3a. The direction of the main beam of an LWA is determined by the phase constant (β) and free-space wavenumber ($k_{0}$ ) according to θ(f) = sin${}^{-1}\left[\beta \left(f\right)/k_{0}\left(f\right)\right]$, where θ is the angle between the broadside and the main beam direction [62]. With a change of frequency, $\beta \left(f\right)/k_{0}\left(f\right)$ changes, and so does θ(f) of an LWA. The directions of the main beam are θ = 39${}^{\circ }$, 42${}^{\circ }$, 57${}^{\circ }$, and 59${}^{\circ }$ away from the broadside, and the half-power beamwidths (HPBWs) are 48${}^{\circ }$, 39${}^{\circ }$, 36${}^{\circ }$, and 27${}^{\circ }$ at 27, 28, 29, and 30 GHz, respectively. This significant change in the HPBW is due to the variation in the antenna’s effective aperture, as the antenna length is quite long. These phenomena are explained using the surface current distributions shown in Figure 4a. It can be seen that as the frequency increases from 27 to 30 GHz, the effective aperture increases, meaning the beam becomes narrower. The directivities are 8, 8.8, 9.2, and 9.9 dBi at 27, 28, 29, and 30 GHz, respectively. With increasing frequency, the HPBW decreases, directivity increases, and the main beam shifts away from the broadside.

### 2.2. Shorter Antenna for Wireless Terminals

For millimeter-wave wireless devices, we need a shorter antenna that takes only a small area of the printed circuit board (PCB) since a wider beam is preferred. It is observed from the surface current distribution in Figure 4a that the antenna radiates most of the power from a short section within the band of our interest. Thus, we can easily reduce the length of the microstrip for our target frequency band. The new length of the microstrip is *L* = 19.2 mm or 1.8$\lambda_{0}$, as shown in Figure 1b. To simplify the antenna design, the far end is terminated using a via at point E in Figure 1b instead of using a coaxial load.

The input reflection coefficient of the LWA (*L* = 1.8$\lambda_{0}$) in Figure 1b is shown in Figure 2. The antenna has an impedance bandwidth of 4.14 GHz (26.49–30.63 GHz), which covers the 28 GHz 5G mobile communication band (27–30 GHz). The surface current distributions (amplitudes) at two different frequencies within the band are shown in Figure 4b. As expected, the effective antenna aperture is long at a higher frequency (30 GHz) compared to that at a lower frequency (27 GHz).

**Figure 5:**
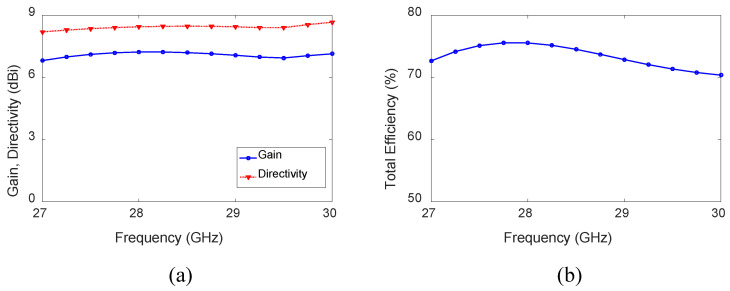
(**a**) Gain and directivity. (**b**) Antenna efficiency of the HW-MLWA of length *L* = 1.8$\lambda_{0}$.

The normalized radiation patterns of the antenna in the x-z plane are shown in Figure 3b. The directions of the main beam are θ = 36${}^{\circ }$, 38${}^{\circ }$, 42${}^{\circ }$, and 62${}^{\circ }$, and HPBWs are 46${}^{\circ }$, 46${}^{\circ }$, 49${}^{\circ }$, and 50${}^{\circ }$ at 27, 28, 29, and 30 GHz, respectively. The HPBWs in the y-z plane are 77${}^{\circ }$, 72${}^{\circ }$, 69${}^{\circ }$, and 60${}^{\circ }$ at 27, 28, 29, and 30 GHz, respectively. The antenna’s effective aperture remains nearly the same because of the shorter antenna length; hence, the antenna’s HPBW does not change significantly. The antenna gain varies between 6.93 and 7.2 dBi, as shown in Figure 5a. The directivity of the antenna varies in the range of 8.3–8.6 dBi, as depicted in Figure 5a. The difference between directivity and gain is due to the losses. Note that increasing the length of the antenna will reduce the difference between the directivity and gain. The total efficiency varies between 76% (27.75 GHz) and 70% (30 GHz) (see Figure 5b) within the band of interest.

## 3. Antenna Radiating at the Broadside

This section describes the design of a 1D LWA radiating at the broadside (θ = 0${}^{\circ }$).

### 3.1. Antenna Configuration

**Figure 6:**
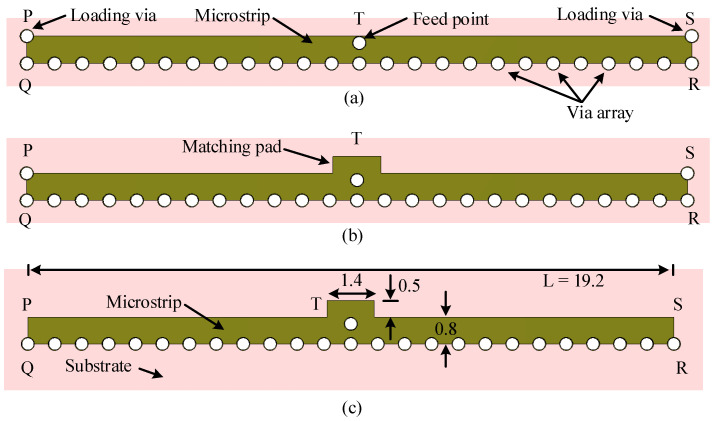
Evolution of the 28 GHz broadside-radiating HW-MLWA. (**a**) Step-1. (**b**) Step-2. (**c**) Step-3. All dimensions are in millimeters.

The broadside radiating LWA was designed on the same FR-4 substrate with the same thickness. The configuration of the broadside radiating antenna is shown in Figure 6c. The microstrip LWA (PQRS) is fed at the center T. The same technique is used to feed the antenna as discussed in Section 2.1. The overall dimensions of the antenna are 19.6 mm × 1.5 mm or 1.83$\lambda_{0}$ × 0.14$\lambda_{0}$.

### 3.2. Antenna Performance

**Figure 7:**
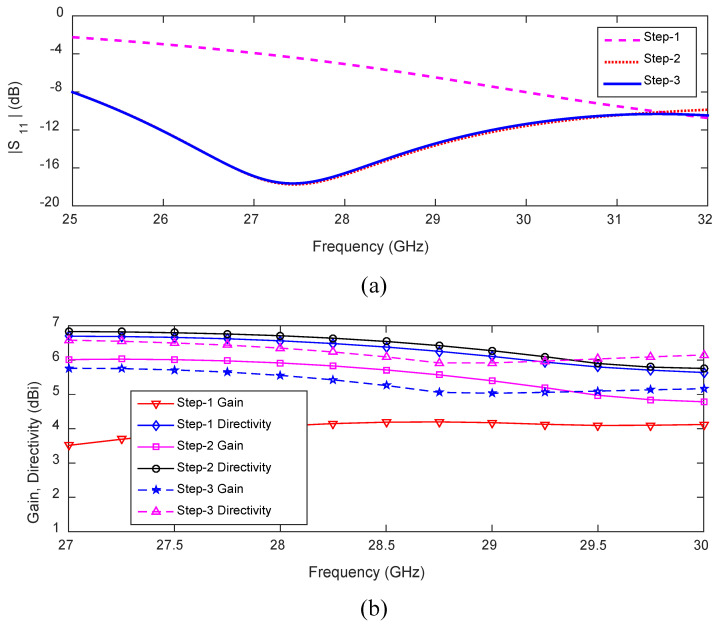
(**a**) Reflection coefficient. (**b**) Gain and directivity corresponding to the three steps in the evolution of the broadside-radiating HW-MLWA.

The design process for this antenna consists of three iterative steps. The antenna design starts with a microstrip LWA, denoted as PQRS, with dimensions of 19.2 mm × 0.8 mm, which has periodically arranged shorting pins along edge QR and two loading vias positioned at corner points P and S, as shown in Figure 6a. The corresponding input reflection coefficient of the antenna is shown in Figure 7a. The antenna of Step 1 has a reflection coefficient below −10 dB in the frequency range of 31.39–32 GHz, which is outside the target frequency band. The gain and the directivity of the antenna vary between 3.8–4.2 dBi and 5.5–6.7 dBi, respectively, as shown in Figure 7b.

In Step 2, a rectangular patch was applied close to the feed point for impedance matching within the targeted band. The dimensions of this matching patch were optimized using a parameter study in CST. The antenna has an input reflection coefficient bandwidth of 25.53–31.73 GHz, which covers the whole 5G millimeter-wave band, as shown in Figure 7a (Step 2 curve). Moreover, the gain and directivity increase in Step 2 compared to Step 1. The improvement of gain is due to better impedance matching in the desired band. The gain improvements at 27.5 and 31 GHz are 2.2 and 0.5 dB, respectively, as shown in Figure 7b. The gain variation within the frequency range of 27–30 GHz is 1.3 dB.

For beam switching, we need to connect feed points at P and S (a detailed discussion is given in the next section) in addition to point T. For this purpose, in Step 3, the two loading vias are removed, and the corresponding reflection coefficient is shown in Figure 7a (Step 3 curve). The antenna reflection coefficient within the frequency range 25–32 GHz is the same as in Step 2. The antenna gain and directivity for Step 3 are shown in Figure 7b and compared with the results of the previous two steps. The gain and directivity variation is less in Step 3 compared to the previous two steps. They are 0.7 and 0.5 dB, respectively.

**Figure 8:**
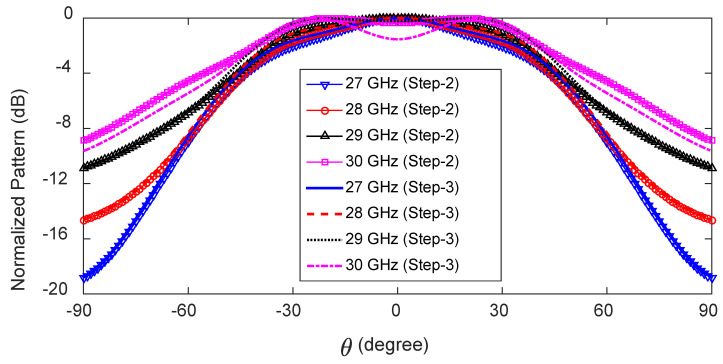
Normalized radiation patterns in the x-z plane of the broadside-radiating HW-MLWA, with and without loading vias.

**Figure 9:**
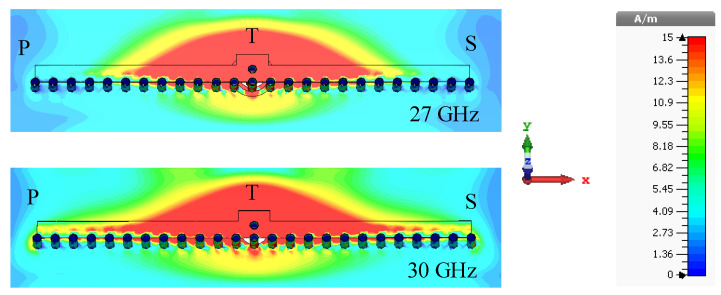
Surface current distributions (amplitude) at 27 and 30 GHz of the broadside-radiating HW-MLWA (Step 3 design).

**Figure 10:**
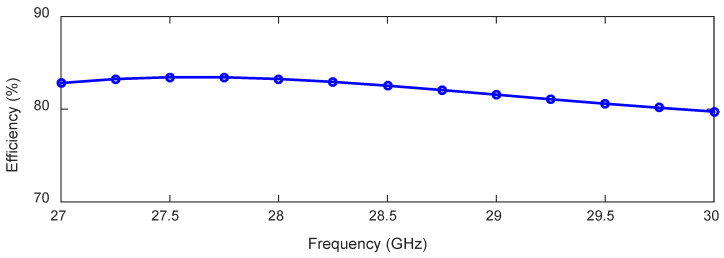
Total efficiency of the 28 GHz broadside-radiating HW-MLWA.

The absence of loading vias has a very negligible effect on the y-z plane radiation patterns within the frequency range of 27–30 GHz. Here, we compare the radiation patterns of the antenna in the frequency range of 27–30 GHz with loading vias (see Figure 6b) and without loading vias (see Figure 6c). In both cases, the antenna radiates a single beam in the frequency range of 27–30 GHz, as shown in Figure 8. The radiation patterns are nearly unaffected in the frequency range of 27–29 GHz when the loading via is removed. This is due to the high leakage rate, i.e., low effective aperture. However, without loading vias, the energy is reflected back from the P and S ends and produces a backward beam at 30 GHz and, hence, the gain along the broadside direction decreases by 1.6 dB (see Figure 8 (30 GHz Step 3 curve)). On the other hand, loading vias absorb the remaining energy and suppress the backward wave. The surface current distributions at 27 and 30 GHz are shown in Figure 9 for the antenna without loading vias (antenna in Figure 6c). The energy reflected back at 30 GHz is higher than the energy reflected back at 27 GHz. The HPBWs of the antenna in the x-z plane are 81${}^{\circ }$, 84${}^{\circ }$, 89${}^{\circ }$, and 92${}^{\circ }$, and in the y-z plane, are 100${}^{\circ }$, 96${}^{\circ }$, 92${}^{\circ }$, and 88${}^{\circ }$ at 27, 28, 29, and 30 GHz, respectively. The antenna efficiency varies between 80% and 84% within the frequency range of 27–30 GHz, as shown in Figure 10.

## 4. 1D Beam Switching

This section presents the design of a 1D beam-switching antenna for 28 GHz 5G wireless terminals, which can point to the beam in three different directions along the antenna axis.

### 4.1. Antenna Configuration

The properties of the previous two antenna designs are incorporated into a single antenna to achieve 1D beam switching using a single radiating element.

**Figure 11:**
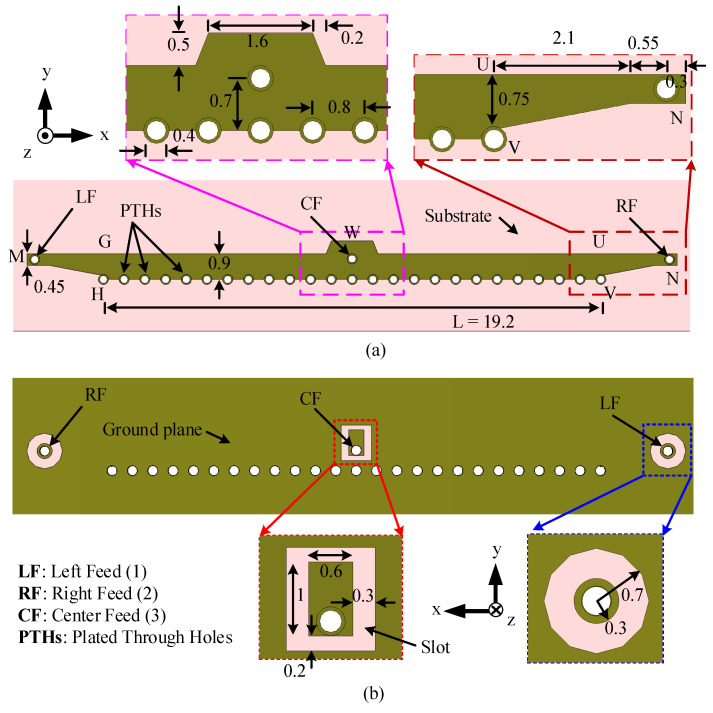
Configuration of the 28 GHz 1D pattern-reconfigurable antenna. (**a**) Top view. (**b**) Back view. All dimensions are in millimeters.

The antenna is fed at three different locations, i.e., left feed (LF), right feed (RF), and center feed (CF), as shown in Figure 11a. To achieve better impedance matching between the coaxial connector and the LWA, two tapered microstrip lines, MG and NU, are used for LF and RF, respectively. A trapezoidal patch is placed at location W to improve the impedance matching for the center feed. In this design, only one feed is ‘ON’ at a time while the other two feeds are ‘OFF’ (worked as matched termination). The feed arrangements for LF and RF are the same as those used in the previous two sections. For CF, a rectangular slot is allowed in the ground plane to ensure isolation between the center pin of the coaxial connector and the ground plane, leaving a rectangular patch at the location of CF. Dimensions of the slot and rectangular patch are given in Figure 11b. The overall dimensions of the antenna are 25.1 mm × 1.6 mm or 2.34$\lambda_{0}$ × 0.15$\lambda_{0}$.

### 4.2. Design Procedure and Working Principle

**Figure 12:**
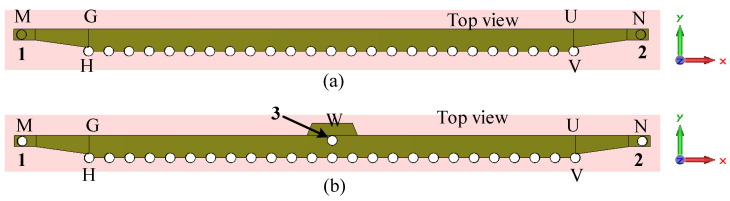
Evolution of the 1D pattern reconfigurable antenna for 28 GHz millimeter-wave 5G wireless terminal. (**a**) Step-1. (**b**) Step-2.

**Figure 13:**
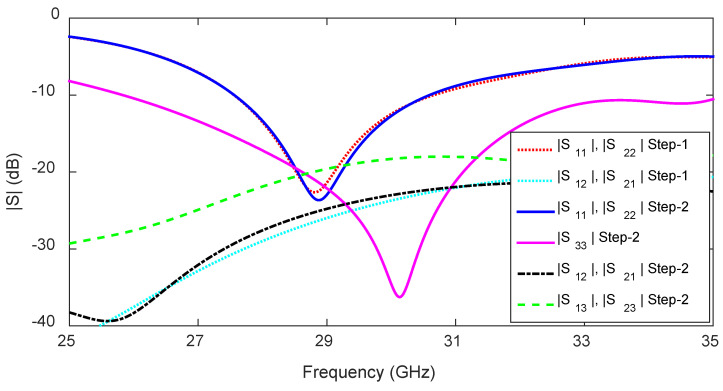
S-parameters corresponding to the two evolution steps of the 1D beam-switching antenna.

The two steps of evolution of the 1D pattern reconfigurable antenna are depicted in Figure 12, and the corresponding S-parameters are shown in Figure 13. In the first step (Step 1), the antenna is fed from either side by activating one port at a time, as shown in Figure 12a. The antenna in Step 1 has a −10 dB reflection coefficient bandwidth of 3.2 GHz (27.5–30.7 GHz) for LF and RF, as shown in Figure 13 (Step 1 curves). The transmission coefficient between the LF and RF feeds is below −23 dB in the operating band. As expected, the antenna in Step 1 is capable of switching the radiation pattern in two different directions. It radiates in the forward (the region between the +z and +x-axes) and backward (the region between the +z and −x-axes) directions when LF and RF, respectively, are ‘ON’.

In Step 2, an arrangement for the center feed is added to the antenna together with a trapezoidal patch, which is aligned to the free edge GU, as shown in Figure 12b. The trapezoidal patch has a significant effect on the antenna input impedance, and its dimensions are optimized using the parameter analysis in CST. The S-parameters for the optimized structure are shown in Figure 13. The impedance bandwidth for CF is 25.8–35 GHz, while that for LF and RF remains unaffected (27.45–30.7 GHz). The transmission coefficients ($|S_{12}|$, $|S_{21}|$) between the LF and RF are always below −24 dB within the −10 dB reflection coefficient bandwidth of the antenna, as shown in Figure 13 (Step 2 curves). Furthermore, the transmission coefficients ($|S_{13}|$, $|S_{31}|$, $|S_{23}|$, $|S_{32}|$) between the LF/RF and CF are always below −18 dB within the −10 dB reflection coefficient bandwidth, as shown in Figure 13 ($|S_{13}\left|,\right|S_{23}|$ Step 2 curve).

**Figure 14:**
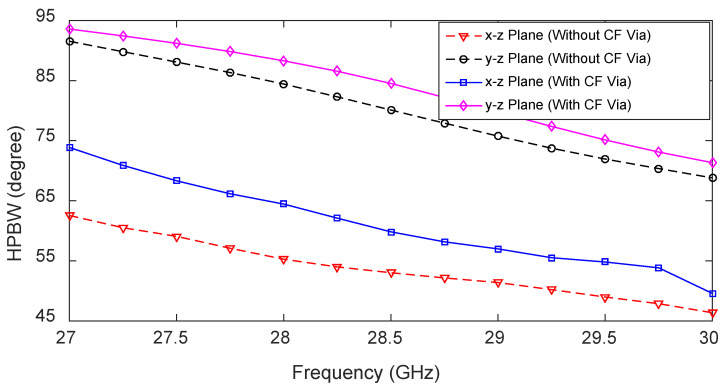
Effects of the center feed (CF) on the HPBW of the 1D pattern reconfigurable antenna when the left feed (LF) is active.

The effect of the CF on the HPBW of the antenna in x-z and y-z planes when LF/RF is ‘ON’ is presented in Figure 14. When the CF is introduced, the beamwidth increases throughout the operating band in both the x-z and y-z planes. Note that when one feed is ‘ON’, the other two feeds act as a matched load, and they are ‘OFF’.

**Figure 15:**
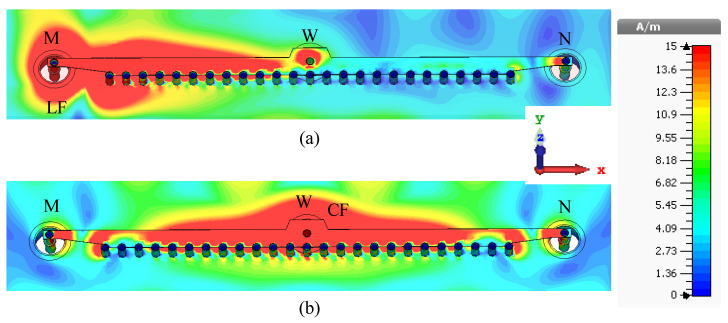
Surface current distributions (amplitude) of the 1D pattern reconfigurable antenna at 29.5 GHz. (**a**) LF is ‘ON’. (**b**) CF is ‘ON’.

When a side feed LF/RF is ‘ON’, the center feed restricts energy traveling towards the other side of the antenna (see Figure 15a). Therefore, the direction of the main lobe shifts slightly toward the broadside and improves the isolation between the two side feeds, LF and RF. Moreover, when LF/RF is ‘ON’, the current is strong between the active feed (LF or RF) and CF. When the CF is ‘ON’, the current is uniformly distributed at the two sides of point W, as depicted in Figure 15b.

**Figure 16:**
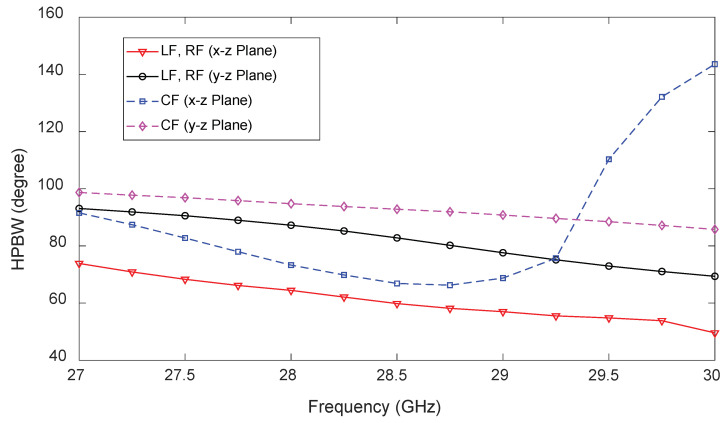
Predicted half-power beamwidth (HPBW) in the x-z and y-z planes of the 1D pattern reconfigurable antenna for CF, LF, and RF.

Figure 16 shows the HPBW of the 1D pattern reconfigurable antenna with LF, RF, and CF being ‘ON’ (one at a time). Note that the HPBW for LF and RF is equal at each frequency point. As expected, the HPBWs for LF and RF in both the x-z and y-z planes reduce gradually with an increase in frequency, which is due to the increase in the antenna’s effective aperture. Similarly, the HPBW of the antenna for CF in the y-z plane reduces gradually with frequency since, with an increase in frequency, the antenna’s effective aperture increases. The HPBW in the x-z plane decreases with an increase of frequency up to 28.5 GHz because two beams from elements WU and WG merge well and, hence, the directivity of the antenna increases. As the frequency increases beyond 28.5 GHz, the merged beam starts to spread, so the HPBW of the antenna increases.

## 5. Measured and Predicted Results

**Figure 17:**
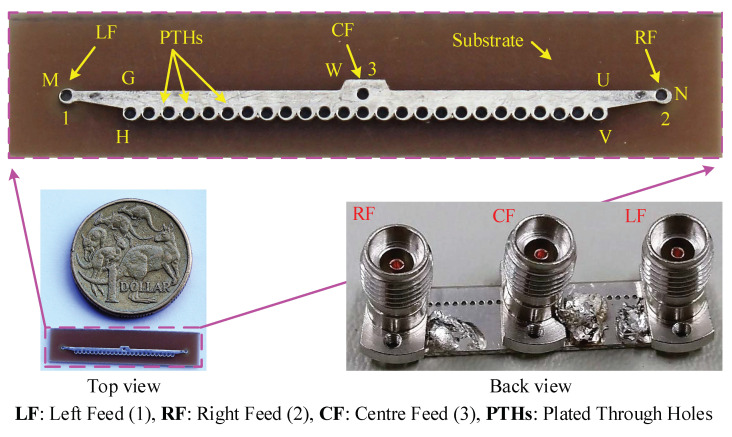
Fabricated 1D pattern reconfigurable LWA for 28 GHz 5G wireless terminals.

To validate the 1D antenna concept, a prototype was fabricated and measured. It is shown in Figure 17. The S-parameters were measured using a vector network analyzer, and the radiation patterns were measured at the Antenna & RF Measurements Facility, Politecnico di Torino, Italy.

### 5.1. S-Parameters

**Figure 18:**
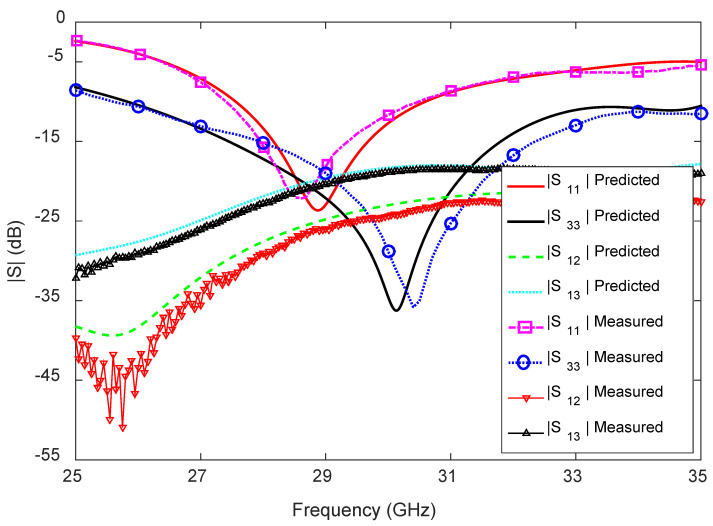
Measured and predicted S-parameters of the 1D pattern reconfigurable antenna for 28 GHz millimeter-wave 5G wireless terminals.

The measured S-parameters of the 1D pattern reconfigurable antenna are shown in Figure 18, together with the predicted ones. Excellent agreement is observed between the measured and the predicted results. The measured −10 dB reflection coefficient bandwidth of the antenna is 3.2 GHz (27.3–30.5 GHz) for the LF, and this is the same for RF and, hence, the results for only LF are presented here. Moreover, the measured −10 dB reflection coefficient bandwidth of the antenna is 9.4 GHz (25.6–35 GHz) for the center feed (CF).

The measured transmission coefficients ($|S_{12}|$, $|S_{21}|$) between the LF and RF are below −24 dB within the −10 dB reflection coefficient bandwidth (27.3–30.5 GHz) of the antenna. Apart from this, the measured transmission coefficients ($|S_{13}|$, $|S_{31}|$, $|S_{23}|$, $|S_{32}|$) between the LF/RF and the CF are below −19 dB, between 25.6 and 35 GHz. Again, excellent agreement is observed between the predicted and measured results for all three feeds, i.e., LF, RF, and CF.

### 5.2. Radiation Performance

**Figure 19:**
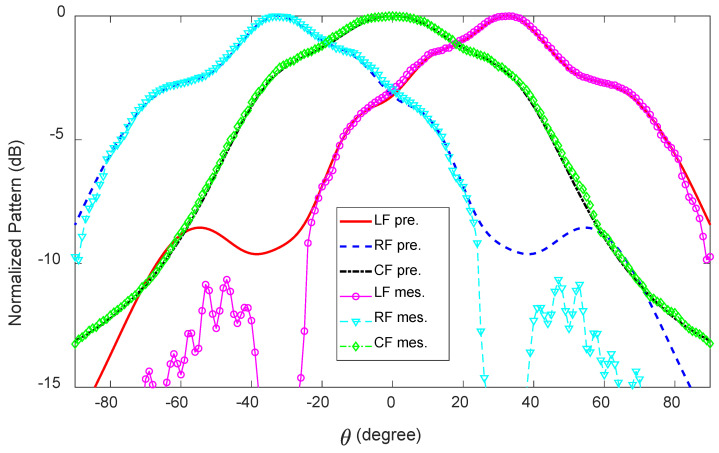
Measured and predicted radiation patterns (normalized) of the 1D pattern reconfigurable antenna in the x-z plane at 28 GHz.

**Figure 20:**
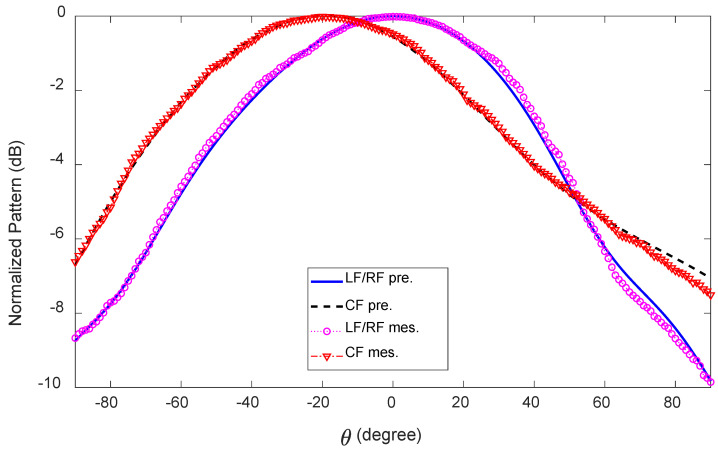
Measured and predicted radiation patterns (normalized) of the 1D pattern reconfigurable antenna in the y-z plane at 28 GHz.

The measured and predicted radiation patterns in the x-z plane at 28 GHz for all three feeds are illustrated in Figure 19. When fed at LF, the measured main lobe directions are θ = 30${}^{\circ }$, 33${}^{\circ }$, 35${}^{\circ }$, 37${}^{\circ }$ at 27, 28, 29, and 30 GHz, respectively, and when fed at RF, they are exactly the same, except negative. For CF, the x-z plane radiation patterns point towards the broadside, and no beam splitting is observed within the frequency range of 27–30 GHz. The measured HPBWs for LF/RF in the x-z plane are 78${}^{\circ }$, 68${}^{\circ }$, 61${}^{\circ }$, and 53${}^{\circ }$ at 27, 28, 29, and 30 GHz, respectively, and for CF, they are 94${}^{\circ }$, 77${}^{\circ }$, 74${}^{\circ }$, 146${}^{\circ }$. Figure 20 shows the measured and predicted y-z plane radiation patterns of the antenna at 28 GHz in the main beam direction when LF/RF and CF are active, one at a time. For LF/RF, the measured HPBWs in the y-z plane are 97${}^{\circ }$, 92${}^{\circ }$, 80${}^{\circ }$, and 73${}^{\circ }$ at 27, 28, 29, and 30 GHz, respectively. For CF, the radiation patterns in the y-z plane point between −20${}^{\circ }$ and −27${}^{\circ }$. In this case, the HPBWs are 102${}^{\circ }$, 98${}^{\circ }$, 95${}^{\circ }$, and 89${}^{\circ }$, respectively, for the above-mentioned frequencies.

**Figure 21:**
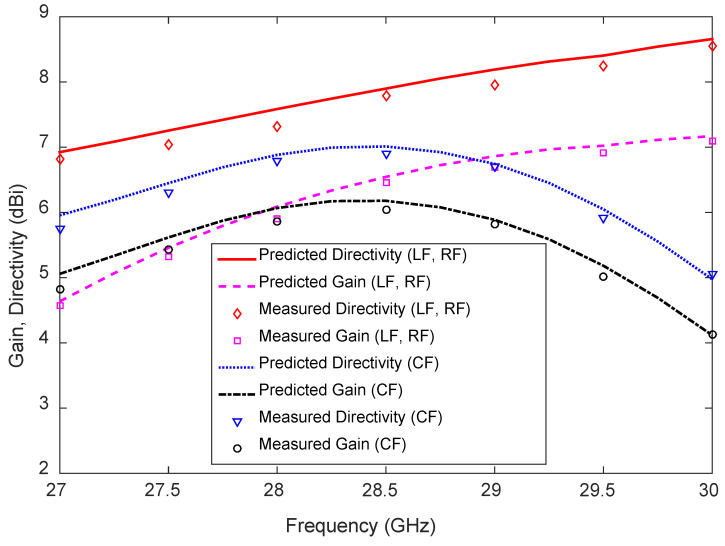
Measured and predicted directivity and gain of the 1D pattern reconfigurable antenna.

The measured and predicted gain and directivity of the 1D pattern reconfigurable antenna are compared in Figure 21; excellent agreement is observed between them. Note that the considerable difference between the directivity and gain is largely attributable to the losses in the dielectric. The measured maximum and minimum directivities of the antenna for LF are 8.6 and 6.8 dBi, respectively, and for CF, they are 6.9 and 5.1 dBi within the frequency range. The directivity of the antenna for LF increases with increasing frequency, but for CF, the directivity increases in the frequency range of 27–28.5 GHz and decreases in the frequency range of 28.5–30 GHz. The measured maximum and minimum gains of the antenna for LF are 7.1 and 4.6 dBi, respectively, and for CF, they are 6.1 and 4.1 dBi, in the frequency range of 27–30 GHz.

**Figure 22:**
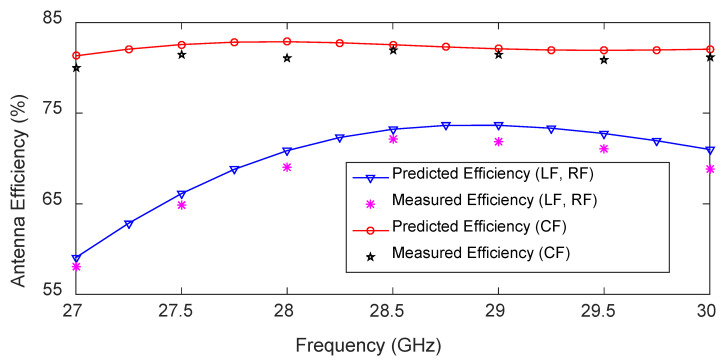
Measured and predicted antenna efficiency of the 1D pattern reconfigurable antenna.

The measured antenna efficiency for LF and CF, together with the predicted results, are depicted in Figure 22. Once again, good agreement is observed between the predicted and measured results. The maximum and minimum measured antenna efficiencies are 72% (at 28.5 GHz) and 58% (at 27 GHz), respectively. At the frequency of 28.75 GHz, the antenna efficiency for CF is 8% higher than the antenna efficiency for LF. The maximum and minimum antenna efficiencies for CF are 82% and 80%, respectively, at 28.5 GHz and 27 GHz, respectively.

## 6. 2D Beam Switching

The antenna presented in Section 4 can switch the beam to three different directions along the x-axis. In this section, two identical antennas are placed at a right angle, aligned with the PCB device edge, to achieve beam switching along two axes (along the x and y-axes). The measured and predicted results are described after integrating the antenna into a device’s PCB board. Note that we chose this arrangement for antennas to leave more space for device circuits.

### 6.1. Antenna Configuration

**Figure 23:**
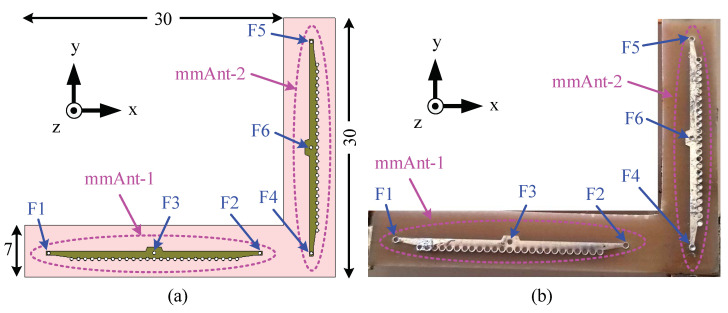
Configuration of the 2D pattern-reconfigurable antenna for the 28 GHz 5G wireless terminal. (**a**) Configuration. (**b**) Prototype. All dimensions are in millimeters.

The configuration of the antenna for 2D beam switching is shown in Figure 23 together with the prototype. Pattern reconfiguration along the x-axis is achieved using the element mmAnt-1 (the corresponding feeds are F1, F2, and F3), and the directions of radiation are as described in the previous section. Additional pattern reconfiguration along the y-axis is achieved using the second element mmAnt-2. The directions of radiation are off-broad-up (the region between the +z and +y axes), off-broad-down (the region between the +z and −y axes), and broadside (along the +z axis) when the feeds F4, F5, and F6 are active, respectively.

### 6.2. Integration into Wireless Device

**Figure 24:**
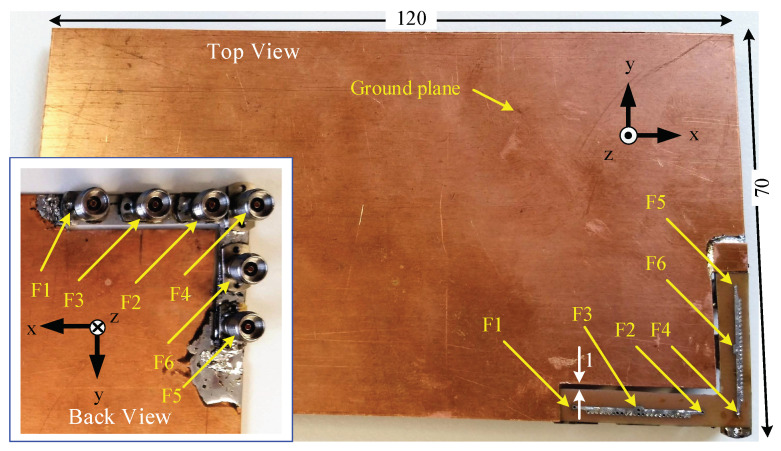
Prototype of the 2D pattern-reconfigurable antenna after integrating into a wireless device’s ground. The inset shows the back view of the antennas with feed points. All dimensions are in millimeters.

The prototype of the 2D pattern reconfigurable antenna after integrating the antenna with a wireless device’s PCB (at a corner) with dimensions of 120 mm × 70 mm is shown in Figure 24. A large antenna ground plane is used to take into account the effect of the system ground on the antenna radiation. A narrow 1 mm gap (by removing PCB) is allowed between the device’s ground plane and the substrate of the antenna to limit any propagation of energy from the antenna to the device’s ground plane.

### 6.3. Measured and Predicted Results

**Figure 25:**
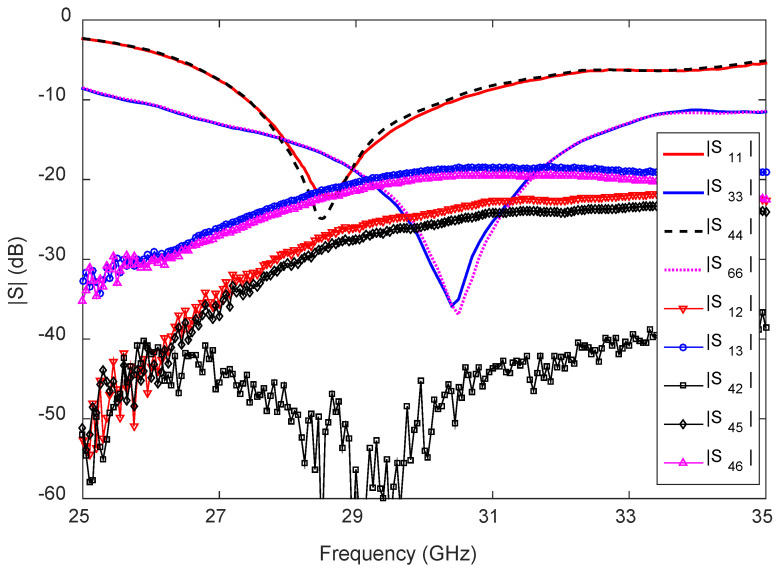
Measured S-parameters of the 2D pattern reconfigurable antenna for the 28 GHz 5G wireless terminal.

From the S-parameter measurements, we found excellent agreement between the predicted and measured results and, hence, for better presentation, only measured results are shown here in Figure 25. The measured −10 dB reflection coefficient bandwidth of the antenna is 3.2 GHz (27.3–30.5 GHz) for the side feeds F1/F2, 2.9 GHz (27.4–30.3 GHz) for the side feeds F4/F5, 9.4 GHz (25.6–35 GHz) for the center feed F3, and 9.3 GHz (25.7–35 GHz) for the center feed F6. The input reflection coefficients $|S_{22}|$, $|S_{44}|$, and $|S_{55}|$ are almost the same as $|S_{11}|$, and the input reflection coefficient is $|S_{66}|$ almost the same as $|S_{33}|$.

The measured transmission coefficients ($|S_{12}|$, $|S_{21}|$) between F1 and F2 are below −24 dB within 27.3–30.5 GHz, and the measured $|S_{45}|$, $|S_{54}|$ are below −27 dB within 27.4–30.3 GHz. Furthermore, the measured transmission coefficients ($|S_{13}|$, $|S_{31}|$, $|S_{23}|$, $|S_{32}|$) between the side feeds (F1/F2) and center feed (F3) of the antenna are below −19 dB between 25.6 and 35 GHz) of the antenna. Meanwhile, the measured $|S_{46}|$, $|S_{64}|$, $|S_{56}|$, $|S_{65}|$ between the side feeds (F4 and F5) and center feed (F6) of the antenna are below −20 dB within the −10 dB reflection coefficient bandwidth. Although the F2 feed of mmAnt-1 and F4 of mmAnt-2 are very close to each other (see Figure 24), the measured $|S_{42}|$ is always below −38 dB within the frequency range 25–35 GHz, indicating that the elements mmAnt-1 and mmAnt-2 do not affect each other’s performance.

**Figure 26:**
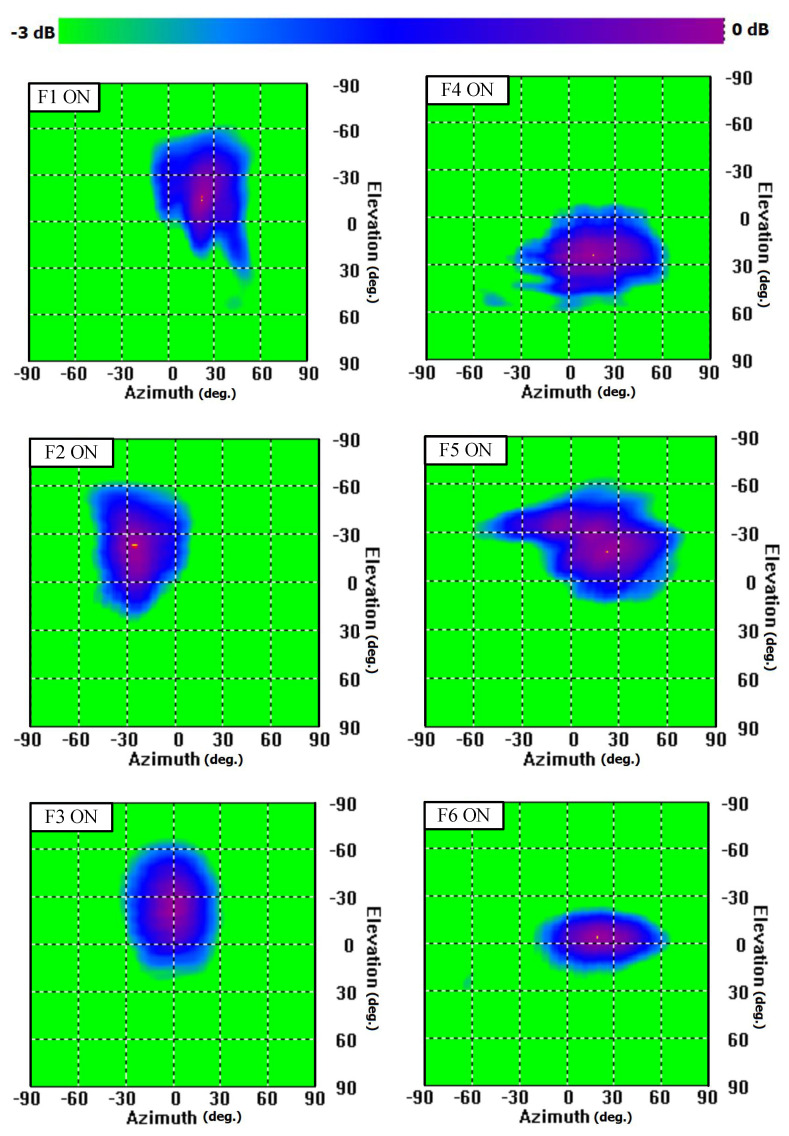
The 2D radiation patterns (normalized) of the 2D pattern-reconfigurable antenna at 28 GHz after integrating the antenna with the ground plane.

The directions of the main lobe of the 2D pattern reconfigurable antenna at 28 GHz are shown in Figure 26, when F1, F2, F3, F4, F5, and F6 are ON (one feed at a time). For better understanding, the radiation patterns are normalized, and the scale depth is set to 3 dB. The nearest green point is the 3 dB point with respect to the peak for each beam. From the 2D radiation patterns, it can be seen that the antenna radiation patterns are slightly tilted from both the x-z and y-z planes after integrating the antenna into the device’s ground plane. This is because the ground plane acts as a reflector for the radiating electromagnetic wave, which is located at one side of the antenna.

At 28 GHz, the radiation pattern of the antenna is in the region between the +x and −y axes, θ = +27${}^{\circ }$, ϕ = −35${}^{\circ }$, when F1 is ON, but the HPBW in the y-z plane is 88${}^{\circ }$, which is wide enough to cover the broadside. The wider HPBW covers a wider area. The radiation patterns of the antenna slowly shift closer to the x-z plane (ϕ = 0${}^{\circ }$) as θ increases in the frequency range 27–30 GHz. Similar behaviors for the radiation patterns of F2 are observed. For F3, the direction of radiation is always aligned with the y-z plane (ϕ = −90${}^{\circ }$), but −24${}^{\circ }$ to −33${}^{\circ }$ tilting along the −y-axis is observed within the frequency range 27–30 GHz. Again, the HPBW in the y-z plane is always above 84${}^{\circ }$, which is sufficient enough to cover the broadside direction, i.e., the +z-axis.

Using the element mmAnt-1, it is possible to scan along the x-axis (ϕ = 0${}^{\circ }$) from θ = +46${}^{\circ }$ to θ = −59${}^{\circ }$. The 3 dB coverage extends to 167${}^{\circ }$ (from θ = +78${}^{\circ }$ to θ = −89${}^{\circ }$) along the x-axis and 103${}^{\circ }$ (from θ = +28${}^{\circ }$ to θ = −75${}^{\circ }$) along the y-axis (ϕ = 90${}^{\circ }$). Using the element mmAnt-2, it is possible to scan along the y-axis (ϕ = 90${}^{\circ }$) from θ = +47${}^{\circ }$ to θ = −39${}^{\circ }$ and along the x-axis (ϕ = 0${}^{\circ }$) from θ = +21${}^{\circ }$ to θ = +30${}^{\circ }$, with a 3 dB coverage of 154${}^{\circ }$ (from θ = +82${}^{\circ }$ to θ = −72${}^{\circ }$) along the y-axis and 99${}^{\circ }$ along the x-axis (from θ = −26${}^{\circ }$ to θ = +73${}^{\circ }$). Note that both antenna elements radiate in the broadside direction.

To summarize, the proposed 2D pattern reconfigurable antenna has a 3 dB coverage of 167${}^{\circ }$ (θ = −89${}^{\circ }$ to +78${}^{\circ }$) in the x-z plane (ϕ = 0${}^{\circ }$), and 154${}^{\circ }$ (θ = −72${}^{\circ }$ to +82${}^{\circ }$) in the y-z plane (ϕ = 90${}^{\circ }$).

## 7. Conclusions

Beam-switching antennas for 28 GHz millimeter-wave 5G wireless devices are presented here. Firstly, an off-broadside (forward) radiating antenna was designed for the 28 GHz millimeter-wave band. Following that, an antenna was designed for radiating in the broadside direction. Using the properties of both antennas, a novel single-element LWA with three ports was developed to achieve 1D beam switching. The 1D beam-switching antenna was prototyped and tested to prove the concept. Using the three feeds, the beam can be pointed at θ = −37${}^{\circ }$ (off-broad-left), θ = 0${}^{\circ }$ (broadside), and θ = +37${}^{\circ }$ (off-broad-right) in the x-z-plane with a combined 3 dB coverage of 128${}^{\circ }$ (θ = −64${}^{\circ }$ to θ = +64${}^{\circ }$) within the frequency range 27–30 GHz. To achieve 2D beam switching, two 1D beam-switching antennas were placed at right angles, at the edge of the PCB device, next to each other. The 2D beam-switching antenna was also prototyped and tested. The 3 dB coverage was 167${}^{\circ }$ (from θ = +78${}^{\circ }$ to θ = −89${}^{\circ }$) in the x-z plane (ϕ = 0${}^{\circ }$) and 154${}^{\circ }$ (from θ = +82${}^{\circ }$ to θ = −72${}^{\circ }$) in the y-z plane (ϕ = 90${}^{\circ }$). This type of antenna can be a low-loss and cost-effective solution for beam switching in 5G wireless terminals operating in the 28 GHz millimeter-wave band.

## Data Availability

Not available.
